# Prediction of Compressive Strength of Fly Ash-Recycled Mortar Based on Grey Wolf Optimizer–Backpropagation Neural Network

**DOI:** 10.3390/ma18010139

**Published:** 2025-01-01

**Authors:** Jing-Jing Shao, Lin-Bin Li, Guang-Ji Yin, Xiao-Dong Wen, Yu-Xiao Zou, Xiao-Bao Zuo, Xiao-Jian Gao, Shan-Shan Cheng

**Affiliations:** 1 School of Architecture and Transportation Engineering, Ningbo University of Technology, Ningbo 315211, China; shaojingjing13@163.com (J.-J.S.); 18958880041@163.com (L.-B.L.); nbutlsjc@126.com (X.-D.W.); 2 School of Safety Science and Engineering, Nanjing University of Science and Technology, Nanjing 210094, China; yxzou0402@gmail.com (Y.-X.Z.); xbzuo@njust.edu.cn (X.-B.Z.); 3 School of Civil Engineering, Harbin Institute of Technology, Harbin 150090, China; gaoxj@hit.edu.cn; 4 School of Engineering, Computing and Mathematics, University of Plymouth, Plymouth PL4 8AA, UK; shanshan.cheng@plymouth.ac.uk

**Keywords:** fly ash-recycled mortar, compressive strength, low water/cement ratio, backpropagation neural network, grey wolf optimizer

## Abstract

The evaluation of the mechanical performance of fly ash-recycled mortar (FARM) is a necessary condition to ensure the efficient utilization of recycled fine aggregates. This article describes the design of nine mix proportions of FARMs with a low water/cement ratio and screens six mix proportions with reasonable flowability. The compressive strengths of FARMs were tested, and the influence of the water/cement ratio (*w*/*c*) and age on the compressive strength was analyzed. Meanwhile, a backpropagation neural network (BPNN) model optimized by the grey wolf optimizer (GWO), namely the GWO-BPNN model, was established to predict the compressive strength of FARM. The input layer of the model consisted of *w*/*c*, a cement/sand ratio, water reducer, age, and fly ash content, while the output layer was the compressive strength. The data set consisted of 150 sets from this article and existing research in the literature, of which 70% is used for model training and 30% for model validation. The results show that compared with the traditional BPNN, the coefficient of determination (*R*^2^) of GWO-BPNN increases from 0.85 to 0.93, and the mean squared error (MSE) of model training decreases from 0.018 to 0.015. Meanwhile, the convergence iterations of model validation decrease from 108 to 65. This indicates that GWO improved the prediction accuracy and computational efficiency of BPNN. The model results of characteristic heat, kernel density estimation, scatter matrix, and the SHAP value all indicated that the *w*/*c* was strongly negatively correlated with compressive strength, while the sand/cement ratio and age were strongly positively correlated with compressive strength. However, the relationship between the contents of fly ash, the water reducer, and the compressive strength was not obvious.

## 1. Introduction

Concrete is the most widely used building material, and its utilization has significant importance in resource conservation and environmental improvement [1]. The effective methods include using recycled aggregates instead of natural aggregates and adding mineral admixtures to replace some cement [2]. The preparation of recycled aggregate concrete (RAC) by using recycled coarse aggregate can not only alleviate the situation of natural resource depletion but also solve the problems of land occupation and environmental pollution caused by the stacking and disposal of construction waste (reducing carbon emissions by 2.175 × 10^5^ KC) [3,4]. However, the residual cement mortar on recycled aggregates with microcracks leads to the inferior performance of RAC compared to ordinary concrete [5]. At present, the main methods to improve the mechanical properties of RAC are to peel off residual mortar or to add mineral admixtures. For the former, physical exfoliation produces a large amount of powder, while chemical or biological treatments are complex, with high economic costs and low environmental benefits [6]. However, using mineral admixtures to replace some cement can reduce carbon emissions (the carbon emissions from cement production account for 5% to 7% of the global total emissions) and also effectively improve the workability of RAC [7]. By adjusting key parameters such as the optimal content, the composite admixture ratio, and the ball milling time of mineral admixtures, the mechanical and durability properties of RAC can also be improved. Therefore, adding an appropriate amount of mineral admixture into RAC is more in line with actual engineering, and fly ash (FA) is currently the most common mineral admixture [8,9], which can reduce carbon emissions and total cost by 22% and 39%, respectively [10]. Research shows that an appropriate amount of FA can effectively improve the slump of concrete [11], reduce the drying shrinkage [12], and increase compressive strength [13]. The addition of FA and other mineral admixtures can also achieve good mechanical properties, while excessive FA can lead to a significant decrease in the mechanical properties of RAC [8]. In summary, after decades of exploration, the preparation technology of RAC with good workability and mechanical properties has become relatively mature [14].

However, a large amount of recycled fine aggregates is generated in the production process of recycled coarse aggregates, accounting for about 40% to 60% of the total mass of waste concrete. A reliable resource utilization approach for recycled fine aggregates is to prepare recycled cement mortar (RCM) [15,16,17]. Compared with recycled coarse aggregate, recycled fine aggregate has a smaller particle size and more residual mortar, resulting in a higher water absorption rate [18]. It leads to a decrease in the effective water when mixing RCM and affects its workability [19]. So, a larger *w*/*c* is required when preparing RCM to ensure its fluidity [20], but leads to a higher porosity of hardened RCM, reducing its mechanical properties and durability [21]. In order to reduce *w*/*c*, it is necessary to increase the amount of water reducer. Meanwhile, similar to RAC, the addition of fly ash is also an effective method to improve the density of RCM and compensate for its mechanical properties [12]. In summary, a lower water/cement ratio (*w*/*c*) and reasonable amounts of FA and water reducer are key parameters to ensure the performance of RCM. In practical engineering, a large number of mixed-proportion experiments are required to determine the precise range of each parameter, ensuring the balance between the fluidity and compressive strength of RCM and meeting the engineering requirements. However, the design of the mixed proportion of RCM is a time-consuming process and limits the promotion of RCM in practical engineering [22]. So, many scholars have proposed a series of empirical formulas and improved Bolomey formulas to establish a quantitative relationship between the mixed proportion and mechanical properties of RCM, achieving the prediction of its compressive strength [23,24]. However, the mixed proportion parameters of RCM are numerous, including a *w*/*c*, sand/cement ratio, aggregate substitution rate, mineral admixture type, and content, etc., and their sensitivity to mechanical properties also varies. Therefore, traditional empirical formulas cannot achieve ideal prediction accuracy [25].

In recent years, with the development of the deep learning theory based on multi-layer neural networks [26], scholars in civil engineering have paid attention to artificial intelligence technologies [27]. Due to their low dependence on mathematical formulas and the ability of autonomous learning to take place based on existing data [28], the theory of deep learning is widely used to predict the performance of cement-based materials (CBMs). For example, the convolutional neural network is good at processing signal data such as images and videos, so it is used for the microstructure feature analysis of CBMs [29]. Recurrent neural network performs well over a continuous time for data processing and is used for the life assessment of CBMs [30]. In terms of predicting the mechanical properties of CBMs, in addition to the methods of linear regression [31], support vector regression [27], and random forest [32], the backpropagation neural network (BPNN) is also a popular intelligent analysis method. Liu et al. [33] used the BPNN to predict the mechanical properties of manufactured sand mortar. Chen et al. [34], Ma et al. [35], Zhong et al. [36], and Tipu et al. [37], respectively, established BPNN prediction models for the compressive strength of fly ash concrete, confined concrete, geopolymer concrete, and recycled concrete. In addition, Yu et al. [38] used the BPNN to predict the carbonation depth of recycled concrete. However, the convergence direction of the BPNN algorithm is the gradient direction of the mean square error (MSE), which is extremely sensitive to the initial correct threshold. The improper selection of initial parameters can lead to a local minimum and slow convergence speed [39]. Therefore, various algorithms are adopted to optimize the BPNN, such as the genetic algorithm [40], particle swarm optimization algorithm [41], differential algorithm [42], artificial ant colony algorithm [43], fruit fly optimization algorithm [44], glowworm swarm algorithm [45], and whale optimization algorithm [46] to avoid falling into local convergence traps. The grey wolf optimizer (GWO) is an animal intelligence optimization algorithm that simulates the cooperative hunting behavior of grey wolf populations [47,48]. Compared with other animal algorithms, the GWO has the characteristics of a simple structure, few adjustable parameters, and easy implementation, and can achieve a balance between local optimization and global searches. So, it exhibits excellent performance in solving accuracy and convergence speeds [49] and is often used to optimize the BPNN.

In this article, fly ash-recycled mortar (FARM) is taken as the research object, aiming to explore the quantitative relationship between its mix proportion and compressive strength. A series of mixed proportions of FARM with low *w*/*c* were designed, and their flowability and compressive strength were tested. Based on fluidity, reasonable mix proportions could be initially determined, and the influence of *w*/*c*, age, and the water reducer on the compressive strength of FARM was further analyzed. Meanwhile, the grey wolf optimizer-backpropagation neural network (GWO-BPNN) model with a topology structure of 5-64-32-1 was established to predict the compressive strength of FARM. The model input layer consisted of a *w*/*c*, cement/sand ratio, water reducer, age, and FA content, while the output layer was the compressive strength. By inputting 150 sets of data (including data tested in this paper and existing data in the literature), the GWO-BPNN model was trained to predict the compressive strength of FACM under different mix proportions. This can provide a basis for optimizing the design of FACM mix proportions.

## 2. Experimental Section

### 2.1. Materials

The cementitious materials in this study are P.O. 42.5 (Ordinary Portland Cement 42.5 grade), mainly produced by Yangchun Cement Co., Ltd. (Yangchun, China) and grade II FA produced by Yulian Power Plant (Zhengzhou, China); the raw material ingredient composition of cement and FA was determined using an X-Ray fluorescence spectrometer. The composition and properties of the cement clinker are shown in Table 1 and Table 2, and the chemical composition and physical properties of FA are shown in Table 3. According to the previous research results [50], we selected a more reasonable particle size distribution of recycled fine aggregate with a 2.82 fineness modulus, as shown in Table 4, and the particle size was reconfigured by manual sieving. The water reducer used was a high-performance polycarboxylate water reducer with a water reduction rate of 28%.

### 2.2. Mix Proportion

The purpose of this experiment is to test the compressive strength of FARM with low *w*/*c* in order to supplement the database of the GWO-BPNN model. A total of 9 mix proportions were set between a *w*/*c* of 0.2 and 0.26. As the *w*/*c* decreased, the amount of water reducer added relatively increased. The proportion of FA in the cementitious material was 30%, and the cement/sand ratio was 0.89. The specific mix proportions of FARM are shown in Table 5. Before preparing the specimens, fresh FARM was mixed to test its workability by using the apparatus of cement mortar fluidity, and the results are also presented in Table 5. A total of 6 mix proportions with reasonable fluidity (within the range of 150 mm~210 mm) were selected to conduct the test of compressive strength. A triple mold of 40 mm × 40 mm × 160 mm was used to produce the three specimens for each mix proportion. After 24 h of FARM preparation, the specimens were demolded and placed into the curing box with a temperature of 25 °C and a humidity of 95%. After curing for 1 day, 3 days, 7 days, and 28 days, the compressive strengths of FARM specimens with different mix proportions were measured.

### 2.3. Compressive Strength

**Figure 1:**
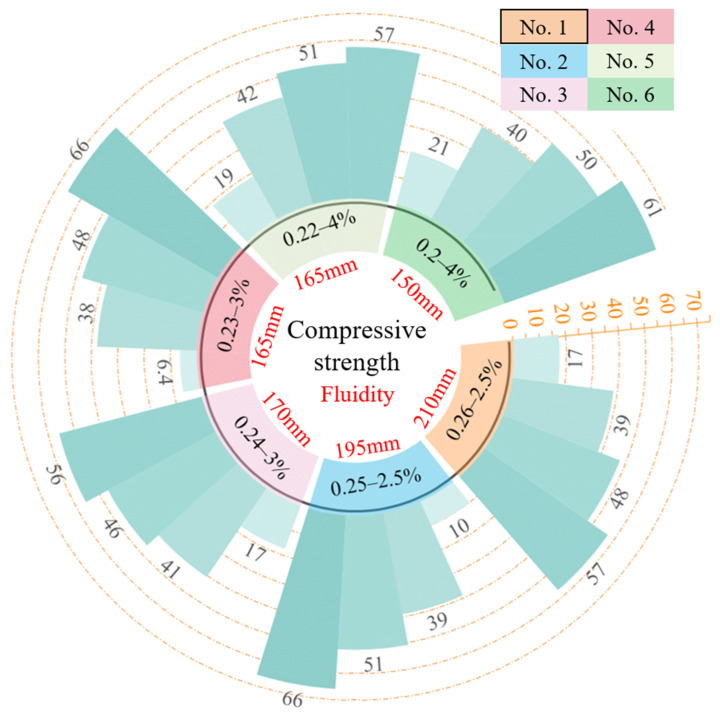
Compressive strength and fluidity of FARM under different mix proportions.

According to the standard method to test cement mortar strength GB/T 17671-2021 [51], six samples of FARM were selected to conduct the compressive strength testing for the different ages of each mix proportion and chose the average value as its compressive strength. If there was a significant difference between the values of one sample and other samples, more samples needed to be added for testing. Figure 1 presents the 1-day, 3-day, 7-day, and 28-day compressive strength of FARM for No. 1–6 mix proportions and their flowability (the average of three tests) but does not provide the compressive strength for No. 7–9. This is because the FARM fluidities of No. 7–9 were low, which is not conducive to the mix of new slurry and could seriously affect its mechanical properties. So, the corresponding strength tests were not conducted. In short, reasonable fluidity is a prerequisite for ensuring the mechanical properties of FARM.

Under the condition of low *w*/*c*, the effect of the water reducer cannot be ignored. It can be seen from Figure 1 and Table 5 that as the water/cement ratio decreases, the effect of the increase in the water reducer content on the fluidity gradually weakens. When the *w*/*c* decreases from 0.26 to 0.25, and the content of the water reducer is 2.5%, the flowability decreases by 15 mm (from 210 mm to 195 mm). When the *w*/*c* decreases from 0.24 to 0.23, and the content of the water reducer increases to 3%, the flowability only decreases by 5 mm (from 170 mm to 165 mm). Comparing the mixed proportions of No. 1 and No. 2 and the compressive strengths of FARMs with the same content of water reducer, the decrease in *w*/*c* leads to an increase in the compressive strength, and the same phenomenon could also be observed between No. 3 and No. 4. However, comparing No. 5 and No. 6, when the *w*/*c* is too small, even though the water reducer content increases to 4% (high level), the compressive strength remains almost unchanged. In addition, the compressive strength of FARM increases rapidly with age, reaching over 80% of its 28-day strength within 7 days.

## 3. Model

### 3.1. BPNN

**Figure 2:**
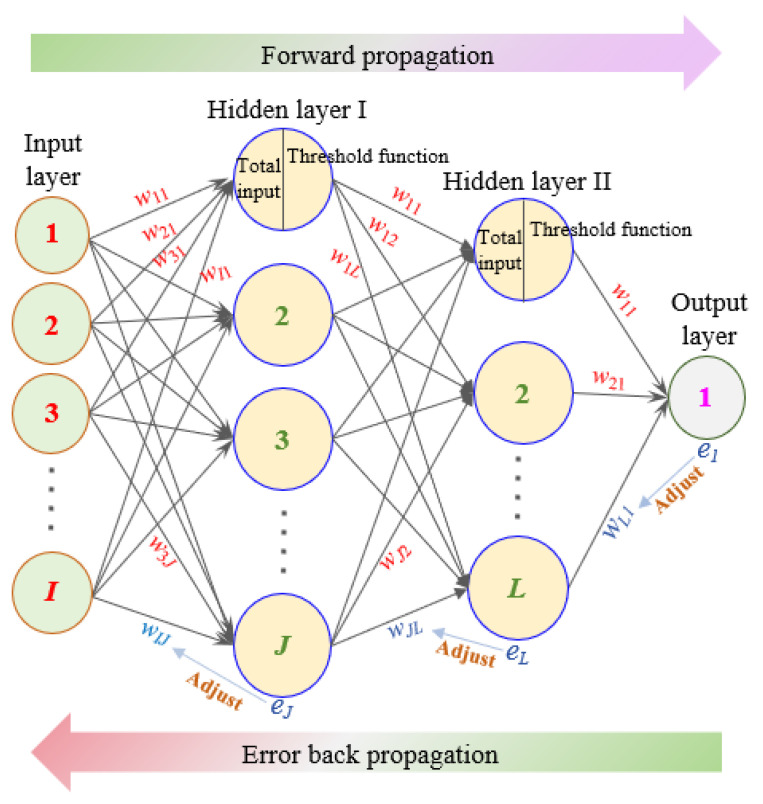
The BPNN structure with 2 hidden layers.

The BPNN belongs to the feedback type deep neural network, which is a computational model that simulates the working mode of the human brain’s nervous system. The neural network in the human brain is composed of groups of neurons, which are the basic units of the nervous system responsible for the input, processing, and output of signals. For the BPNN, it consists of an input layer, one or more hidden layers, and an output layer, and its operation can be divided into two stages [52], as shown in Figure 2. The first stage is the forward propagation of the signal, passing through the hidden layer from the input layer and finally reaching the output layer. The input and output layers are only used for storing input and output values. The hidden layer receives inputs with different weights (*w*), processes them through activation functions (*f*) and biases (*θ*), and outputs them to the next hidden or output layer with different weights. The mathematical formula for the above process can be expressed as follows:

(1) $$\left\{\begin{array}{l} H_{j}^{1}\left(x_{i}\right)=f\left(\sum_{i=1}^{I}w_{ij}\cdot x_{i}+\theta_{j}\right),\;j=1,\;2,\;\ldots ,\;J\\ H_{l}^{2}\left(H_{j}^{1}\right)=f\left(\sum_{j=1}^{J}w_{jl}\cdot H_{j}^{1}+\theta_{l}\right),\;l=1,\;2,\;\ldots ,\;L\\ O_{m}\left(H_{l}^{2}\right)=\sum_{l=1}^{L}w_{lm}\cdot H_{l}^{2}+\theta_{m},\;m=1 \end{array}\right.$$

where *I*, *J*, and *L* represent the number of neurons in the input layer, hidden layer I, and hidden layer II. $x_{i}$ is the value of *i*-th neuron in the input layer. $H_{j}^{1}$ is the output value of the *j*-th neuron in hidden layer I, which serves as the input value for hidden layer II after the processing of the activation function $f\left(a\right)$ and bias θ. $H_{l}^{2}$ is the output value of the *l*-th neuron in hidden layer II. $f\left(a\right)$ is the activation function, which runs on neurons in the BPNN and is responsible for mapping the input to the output. The sigmoid function is often used as an activation function, expressed as Equation (2).

(2) $$f\left(a\right)=\frac{1}{1+e^{\left(-a\right)}},\;\partial f\left(a\right)/\partial a=f\left(a\right)\cdot \left[1-f\left(a\right)\right]$$

The second stage is the backpropagation of errors. When the error *e_m_* between the result of the output layer (*O_m_*) and the target value (*P*_m_) is large, the weight (*w*) and bias (*θ*) need to be adjusted from the output layer to the input layer sequentially. Its mathematical expression is shown in Equation (3).

(3) $$\left\{\begin{array}{l} {w^{\prime }}_{lm}=w_{lm}-\eta \frac{\partial E}{\partial w_{lm}},\;{w^{\prime }}_{jl}=w_{jl}-\eta \frac{\partial E}{\partial w_{jl}},\;{w^{\prime }}_{ij}=w_{ij}-\eta \frac{\partial E}{\partial w_{ij}}\\ {\theta^{\prime }}_{m}=\theta_{m}-\eta \frac{\partial E}{\partial \theta_{m}},\;{\theta^{\prime }}_{l}=\theta_{l}-\eta \frac{\partial E}{\partial \theta_{l}},\;{\theta^{\prime }}_{j}=\theta_{j}-\eta \frac{\partial E}{\partial \theta_{j}}\\ e_{m}=\frac{1}{2}{\left(P_{m}-O_{m}\right)}^{2} \end{array}\right.$$

in which $w^{\prime }$ and $\theta^{\prime }$ are the updated weight and bias, calculated by Equation (4).

(4) $$\left\{\begin{array}{l} {w^{\prime }}_{lm}=w_{lm}+\eta H_{l}^{2}e_{m},\;{w^{\prime }}_{jl}=w_{jl}+\eta H_{l}^{2}\left(1-H_{l}^{2}\right)H_{j}^{1}w_{lm}e_{m},\;{w^{\prime }}_{ij}=w_{ij}+\eta H_{j}^{1}\left(1-H_{j}^{1}\right)x_{i}w_{jl}e_{m}\\ {\theta^{\prime }}_{m}=\theta_{m}+e_{m},\;{\theta^{\prime }}_{l}=\theta_{l}+\eta H_{l}^{2}\left(1-H_{l}^{2}\right)w_{lm}e_{m},\;{\theta^{\prime }}_{j}=\theta_{j}+\eta H_{j}^{1}\left(1-H_{j}^{1}\right)w_{ij}e_{m} \end{array}\right.$$

When the error *e_m_* is less than the set value or the maximum number of iterations is reached, the above neural network stops running; otherwise, it returns to the first stage. In the above process, the excessive number of hidden layers and neurons leads to overfitting, while the insufficient hidden layers increase errors. So, the number of hidden layers is always determined by the trial-and-error method. As for the number of neurons in the hidden layer, this is initially determined by Equation (5) and can be continuously adjusted by the trial-and-error method until the optimal framework of BPNN is found.

(5) $$N_{h}=\frac{N_{s}}{c\left(N_{x}+N_{y}\right)}$$

where *N_s_* is the number of data samples. *N_x_* and *N_y_* represent the number of input layers and output layers, respectively. *c* is a constant, usually chosen as a single digit.

### 3.2. GWO

As described above, the BPNN is very sensitive to initial weights and biases [53] and is prone to falling into the dilemma of local minima. The GWO is an intelligent optimization algorithm with a global search capability that can solve the above problems. The GWO simulates the leadership and hunting levels of grey wolves in nature. There are four roles in the wolf pack. The *α* wolf is responsible for leading the pack and can keenly perceive the position of prey. The *β* wolf can perceive the position of prey. The *δ* wolf is responsible for assisting the *α* and *β* wolves, and the *ω* wolf is responsible for chasing prey. In the selection process, the grey wolf will adjust according to its adaptation situation, and the grey wolf with the strongest adaptability will be updated as the *α* wolf, whose position coordinates are the global optimal solution [54]. The GWO mainly involves two steps, namely surrounding prey and hunting prey, and the surrounding algorithm constructed by Mirjalili et al. [48] is shown in Equations (6) and (7).

(6) $$\vec{D}\left(t\right)=\left|\vec{C}\cdot {\vec{X}}_{p}\left(t\right)-\vec{X}\left(t\right)\right|,\;\vec{X}\left(t+1\right)={\vec{X}}_{p}\left(t\right)-\vec{A}\vec{D}\left(t\right)$$

(7) $$\vec{A}=2\vec{a}\cdot {\vec{r}}_{1}-\vec{a};\;\;\vec{C}=2{\vec{r}}_{2};\;\;\vec{a}\left(t\right)=2\cdot \left(1-\frac{t}{M}\right)$$

where *t* represents the current iterations. ${\vec{X}}_{P}$ is the position vector of the prey (the initial weights and biases that need to be optimized in BPNN, i.e., the optimization objective). $\vec{X}\left(t+1\right)$ represents the position vector of the grey wolf after the *t* + 1 update. $\vec{D}$ represents the distance between the grey wolf and its prey (the error between weight, bias, and optimization objective). ${\vec{r}}_{1}$ and ${\vec{r}}_{2}$ are the random vectors generated in [0, 1]. $\vec{a}$ is a linear convergence factor that decreases from 2 to 0 as the iterations increase. *M* is the maximum number of iterations. $\vec{A}$ is a random vector that changes with $\vec{a}$. When $\vec{a}\geq 1$, $\left|\vec{A}\right|\geq 1$. When $\vec{a}<1$, $\left|\vec{A}\right|<1$. C is a random vector between [0, 2], representing the random weight of the influence of the grey wolf’s position on the prey’s position to enhance the algorithm’s global search ability and robustness [55].

**Figure 3:**
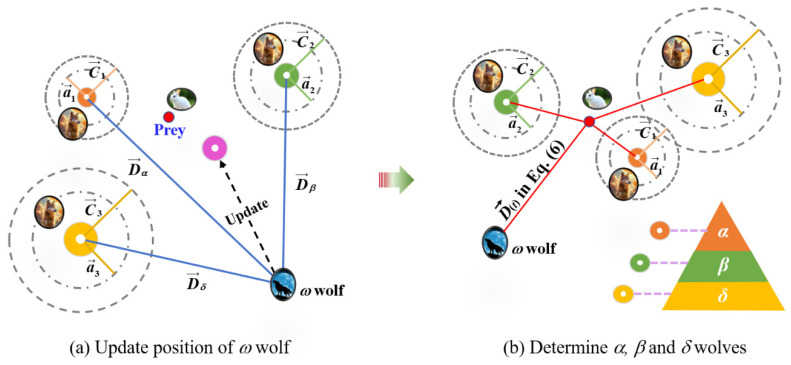
The GWO diagram.

Through the calculation of the surrounding algorithm Equations (6) and (7), the positions of the wolves can be obtained. The three optimal solutions are selected to become the positions of the *α*, *β,* and *δ* wolves in the hunting algorithm, which have potential location information of the prey and can guide the *ω* wolf to search for prey, that is, update the positions of the *ω* wolve, as shown in Figure 3. The above is the process of hunting prey, and its mathematical expressions are shown in Equations (8)–(10).

(8) $${\vec{D}}_{\alpha }=\left|{\vec{C}}_{1}\cdot {\vec{X}}_{\alpha }-\vec{X}\right|;\;{\vec{D}}_{\beta }=\left|{\vec{C}}_{2}\cdot {\vec{X}}_{\beta }-\vec{X}\right|;\;{\vec{D}}_{\delta }=\left|{\vec{C}}_{3}\cdot {\vec{X}}_{\delta }-\vec{X}\right|$$

(9) $${\vec{X}}_{1}={\vec{X}}_{\alpha }-{\vec{A}}_{1}\cdot \left({\vec{D}}_{\alpha }\right);\;{\vec{X}}_{2}={\vec{X}}_{\beta }-{\vec{A}}_{2}\cdot \left({\vec{D}}_{\beta }\right);\;{\vec{X}}_{3}={\vec{X}}_{\delta }-{\vec{A}}_{3}\cdot \left({\vec{D}}_{\delta }\right)$$

(10) $$\vec{X}\left(t+1\right)=\frac{{\vec{X}}_{1}+{\vec{X}}_{2}+{\vec{X}}_{3}}{3}$$

in which $\vec{X}$ is the position vector of the *ω* wolf. ${\vec{X}}_{\alpha }$, ${\vec{X}}_{\beta }$, and ${\vec{X}}_{\delta }$ are the position vectors of *α*, *β,* and *δ* wolves. ${\vec{D}}_{\alpha }$, ${\vec{D}}_{\beta }$, and ${\vec{D}}_{\delta }$ represent the distances between the *ω* wolf and the three optimal grey wolves. *C*_1_, *C*_2_, *C*_3_, *A*_1_, *A*_2_, and *A*_3_ are random vectors.

Substituting $\vec{X}\left(t+1\right)$ obtained from the hunting algorithm Equation (10) into the surrounding algorithm Equations (4) and (5), a new round of iteration is conducted to update the positions of the *α*, *β,* and *δ* wolves (${\vec{X}}_{\alpha }$, ${\vec{X}}_{\beta }$ and ${\vec{X}}_{\delta }$) and then update the position of the *ω* wolve (${\vec{X}}_{\omega }$). Through the continuous iteration reaching the specified number of iterations, the latest position of the *α* wolf is taken as the optimal solution, which contains the optimized initial weights and biases in the BPNN.

### 3.3. Model Framework

**Figure 4:**
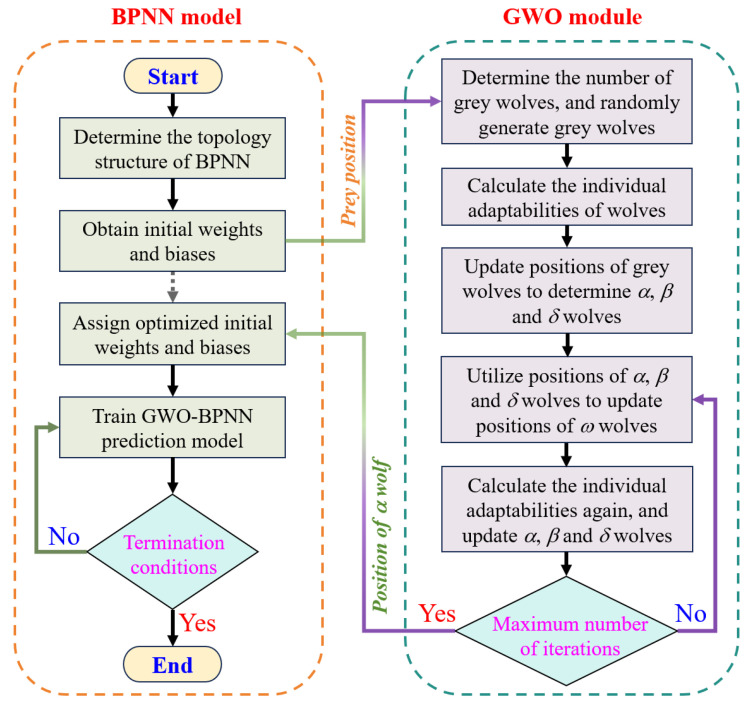
Flowchart of the GWO-BPNN model.

Based on the above theory, a BPNN model optimized by the GWO, namely the GWO-BPNN model, is established. Its self-learning ability is used to carry out the prediction training of FARM compressive strength. Many scholars have demonstrated the optimization performance of the GWO on the BPNN. Tian et al. [49] reduced the optimized model error by 14%, and Guo et al. [56] likewise improved the accuracy of the real model by 6% through GWO optimization. In this paper, the topology structure of the BPNN is 5-64-32-1. The input layer consists of the *w*/*c*, cement/sand ratio, age, water reducer, and FA content, and the output layer is the 28-day compressive strength of FARM. On the basis of considering the computational efficiency of the model, the number of neurons in two hidden layers was determined to be 64 and 32 by the trial-and-error method, respectively. The combination structure of “more” and “less” neurons can improve the convergence and accuracy of the model. In the GWO module, five grey wolves and 200 iterations were set to optimize the initial weights and biases of BPNN. The training and prediction process of GWO-BPNN is shown in Figure 4.

As shown in Figure 4, the role of the GWO is to adjust the appropriate initial weights (*w*) and biases (*e*). After randomly providing the initial weight and bias in the BPNN model, they form ${\vec{X}}_{P}\left(w,\;e\right)$ in the GWO module. According to the randomly given positions of grey wolves $\vec{X}$ and $\vec{D}$ can be calculated through Equations (6) and (7) to determines three optimal solutions ${\vec{X}}_{\alpha }\left(w,\;e\right)$, ${\vec{X}}_{\beta }\left(w,\;e\right)$ and ${\vec{X}}_{\delta }\left(w,\;e\right)$, namely the position of the *α*, *β,* and *δ* wolves. Then, the position of *ω* wolves ${\vec{X}}_{\omega }\left(w,\;e\right)$ can further be updated as Equations (8)–(10). So, the $\vec{D}$ can be calculated again using ${\vec{X}}_{\alpha }\left(w,\;e\right)$, ${\vec{X}}_{\beta }\left(w,\;e\right)$, ${\vec{X}}_{\delta }\left(w,\;e\right)$, and ${\vec{X}}_{\omega }\left(w,\;e\right)$ to determine the optimal solution. After a specified number of iterations, the first optimal solution ${\vec{X}}_{\alpha }\left(w,\;e\right)$, namely the position of the *α* wolf, is returned to the BPNN model as the initial weight and bias.

## 4. Data Sources and Evaluating Indicator

In various models of machine learning, when the number of data is small, it is common to divide the data into a 70% training set and a 30% validation set [57,58,59]. As the amount of data becomes larger, the proportion of the training set can be increased [60]. Therefore, in this paper, a total of 150 data sets were collected, and the data division of 7:3 was used. The data sources are shown in Table 6. The input and output data in the GWO-BPNN are standardized to eliminate the order of magnitude differences [61], as shown by Equation (11), improving the accuracy of prediction results.

(11) $${x^{\prime }}_{ij}=\frac{x_{ij}-\mu_{i}}{\sigma_{i}}\;j=1,\;2\;\ldots ,\;m;\;\mu_{i}=\frac{1}{m}\sum_{j=1}^{m}x_{ij};\;\sigma_{i}=\sqrt{\frac{1}{m}\sum_{j=1}^{m}{\left(x_{ij}-\mu_{i}\right)}^{2}}$$

where $x_{ij}$ is the *j*-th value of the *i*-th data vector ($X_{i}=\left[x_{i1}\;x_{i2}\;\ldots \;\;x_{ij}\;\ldots \;\;x_{im-1}\;\;x_{im}\right]$). *i* is the data type and $i=\left[1,\;2\;\ldots ,\;6\right]$. *m* is the data set and *m* = 150. ${x^{\prime }}_{ij}$ is the standardized data value of $x_{ij}$. $\mu_{i}$ and $\sigma_{i}$ are the mean value and standard deviation of the *i*-th type data.

The coefficient of determination (*R*^2^) and the loss function (MSE) were chosen as the evaluating indicators to analyze the performance of the GWO-BPNN model. *R*^2^ reflects the effectiveness of the model’s fit, and MSE reflects the difference between model prediction results and experimental test data, and their expressions are written as Equation (12). When *R*^2^ = 1 and MSE = 0, the predicted values of the model are accurately aligned with the experimental test data, indicating that the accuracy of the GWO-BPNN model reaches 100%.

(12) $$R^{2}=1-\frac{\sum_{i=1}^{m}{\left(P_{i}-O_{i}\right)}^{2}}{\sum_{i=1}^{m}{\left(P_{i}-\bar{P}\right)}^{2}},\;MSE=\frac{1}{m}\sum_{i=1}^{m}{\left(P_{i}-O_{i}\right)}^{2}$$

where is *m* = 150. $O_{i}$ and $P_{i}$ represent the output and the target values. $\bar{P}$ is the average target value of all the test samples.

## 5. Results and Analysis

### 5.1. Performance Analysis

**Figure 5:**
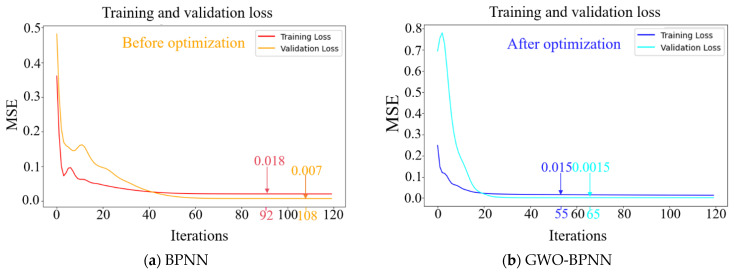
MSE of BPNN prediction models before and after optimization by the GWO.

**Figure 6:**
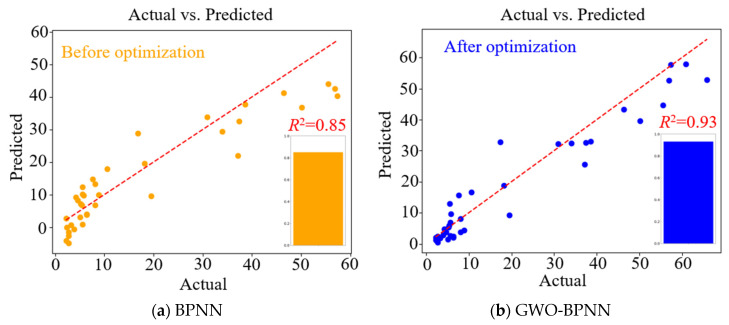
Prediction results of compressive strength and *R*^2^ before and after optimization.

Figure 5 shows the MSE of the BPNN prediction model before and after optimization by the GWO. In addition to assessing the accuracy of the model, the MSE graph can provide a visual understanding of model training. When the training loss continues to decrease, and the gap between the validation loss and the training loss continues to widen, the model is in an overfitting state. It indicates that there is a significant difference between the training data and validation data in the training process, resulting in the GWO-BPNN model performing well in the training process but poorly in the validation process. It can be seen from Figure 4 that the prediction results before and after model optimization converge before 120 iterations without overfitting. The validation loss of the BPNN model reached its optimum at 108 iterations with an MSE of 0.007, while the validation loss of the GWO-BPNN model reached its optimum at 65 iterations with an MSE of 0.0015. The latter has better computational accuracy and efficiency than the former. Figure 6 shows the predicted results of the compressive strength of FARM and *R*^2^ before and after model optimization. It can be seen from Figure 6 that the actual data of the samples are closer to the predicted line after optimization, and the *R*^2^ increased from 0.85 to 0.93, which reflects the fact that the GWO effectively improves the prediction accuracy of the BPNN model.

The prediction values of the GWO-BPNN model, experimental test values, and relative error of FARM compressive strength are shown in Table 7. In the table, the date of No. 1-11 is derived from existing studies, while the date of No. 12–14 is from the validation data in this article. The relative errors between model-predicted values and experimental test values are mostly within 6.5%. So, the GWO-BPNN model can effectively predict the compressive strength of FARM. It should be pointed out that the relative error is relatively large in predicting early strength, reaching up to 11%. This is because the addition of fly ash causes a decrease in the early compressive strength of cement mortar, and the different content of fly ash results in different reduction effects. So, the data gap of early strength from our experiment and studies [62,63,64,65,66,67,68,69] with different contents of fly ash is significant, causing a high error in the model. However, the overall error of this model is still very small (<15%) [14,70], indicating good prediction accuracy.

### 5.2. Visualization Analysis

**Figure 7:**
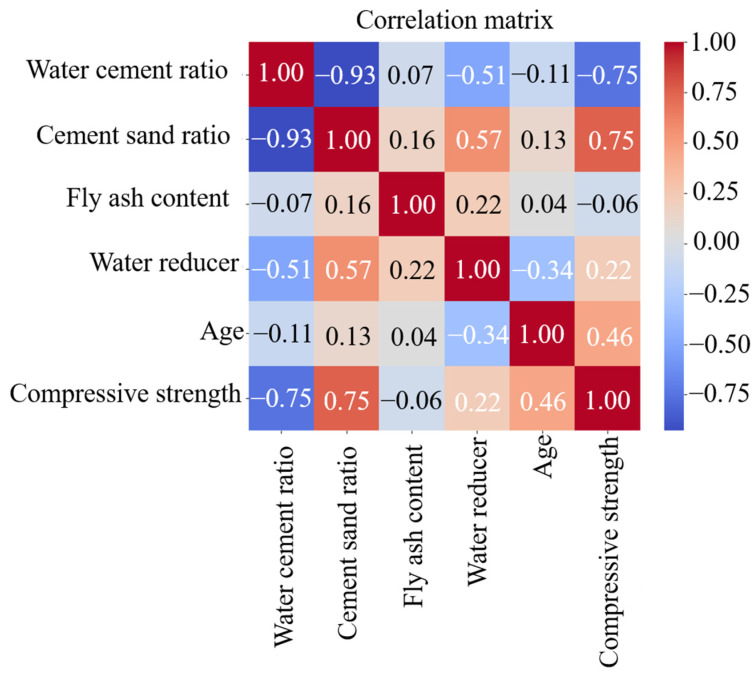
Feature heatmap: Pearson correlation coefficient among various variables.

An advantage of the neural network model lies in utilizing visual data to analyze prediction results [71]. In this paper, a feature heatmap is adopted to display the Pearson correlation coefficient between input and output variables, as shown in Figure 7. The correlation coefficient between the compressive strength of FARM and its water/cement ratio (*w*/*c*) is −0.75, indicating their strong negative correlation, which is consistent with that of ordinary cement mortar (OCM). In the case of high *w*/*c* (greater than 0.6), the compressive strength decreases significantly with the increase in w/*c*, and the strength loss can reach up to 50% [67]. For ordinary *w*/*c* (0.3~0.6), the compressive strength first increases and then decreases with the increase in w/*c*, and the final strength loss reaches 20% [72]. For FARM with a low *w*/*c* (less than 0.3), its compressive strength is higher than that with ordinary *w*/*c* at the same age, especially in the early stage (3 d), where the former is more than twice as high as the latter [73].

The correlation coefficient between the compressive strength and cement/sand ratio is 0.75, indicating their strong positive correlation. However, the correlation between the compressive strength and FA content is −0.06, indicating that the relationship between the two is not clear. Wang et al.’s [74] research shows that when the age of research is over 14–28 days, the compressive strength of FARM with a 30% FA content exceeds that of OCM. This is because the pozzolanic effect of FA is exerted as age increases, which promotes the compaction of a FARM microstructure and thus improves its compressive strength [75]. However, when the FA content exceeds 50%, the compressive strength of FARM is lower than that of OCM [76]. This is because fly ash has low activity, and its large addition reduces the overall hydration rate of FARM. Meanwhile, the reduction in cement also leads to a decrease in the amount of C-S-H gel, and the strength loss caused by this is difficult to compensate for through the pozzolanic effect.

**Figure 8:**
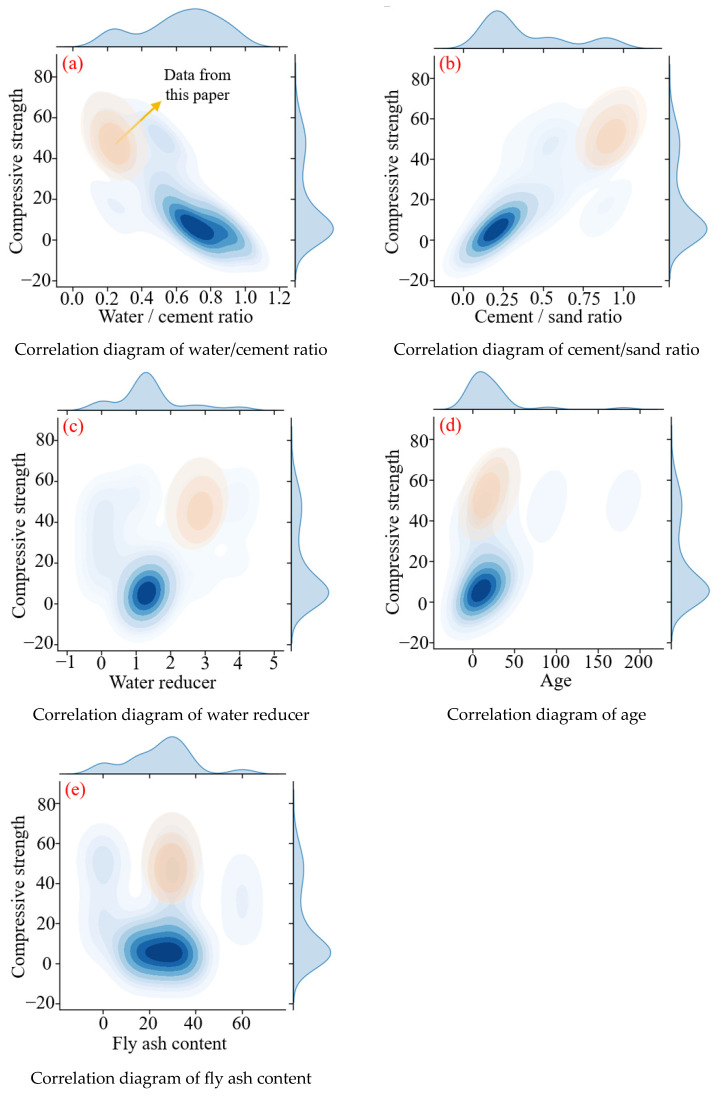
Joint plot of variables—compressive strength.

Another method used for interpreting the data is a joint plot, as shown in Figure 8. The depth of the colors in the graph represents the probability density of the data, and the darker the color, the higher the concentration of the data in that interval [77]. The upper and right sides of the graph show the distribution of independent and dependent variables, respectively. Taking Figure 8a as an example, it can be seen that the compressive strength data are mainly concentrated below 20 MPa, while the corresponding data of *w*/*c* are mainly concentrated between 0.6 and 0.9. This also indicates that most scholars mainly focus on the mechanical properties of FARM with high *w*/*c*, and the data with low *w*/*c* from this article effectively fills this gap in the FARM research. It can also be seen from Figure 8a that when the *w*/*c* decreases from 0.7 to 0.4, the compressive strength rapidly increases. Subsequently, as the *w*/*c* further decreases, the compressive strength no longer significantly improves.

**Figure 9:**
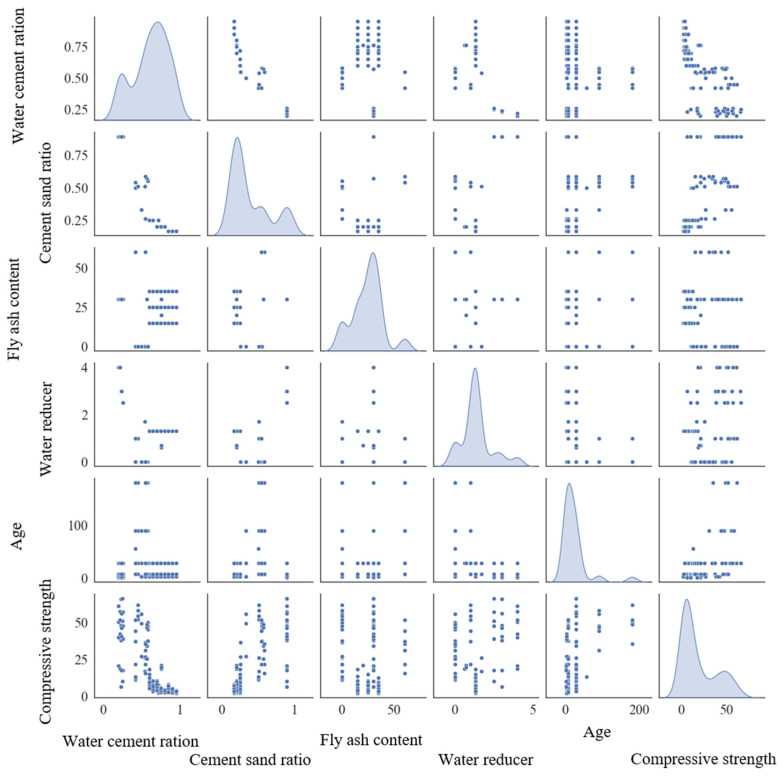
Scatter matrix diagram.

Figure 9 is a scatter matrix diagram that reveals the relationships between different types of data (including the input and output data) and analyzes the influence of input data on compressive strength. In the figure, the density map inserted along the diagonal visualizes the distribution of each data feature [78]. It can be seen from Figure 9 that most studies focus on FARM with a *w*/*c* greater than 0.5, and their compressive strength is lower than 20 MPa. This is due to the porous structure of recycled fine aggregate, which leads to its high water absorption. It is necessary to increase the *w*/*c* to prepare FARM with a 100% replacement rate of recycled fine aggregate, but this results in low compressive strength. Most cement/sand ratios of FARM are less than 0.5, and its compressive strength is lower than 25 MPa. The reason for this phenomenon is that a low cement/sand ratio means an increase in the relative amount of recycled fine aggregate with residual mortar and a decrease in cement content for the bonding effect, resulting in low compressive strength. For FA, its content rarely exceeds 50%, usually between 20% and 30%. FA with smooth spherical particles plays a role in ball lubrication in FARM, effectively reducing water consumption [79]. In addition, the content of the water reducer in FARM usually does not exceed 5%, which seems to be a critical value of the total amount of cementitious material. If exceeding this critical value, the effect of the water reducer is no longer improved and may even have a negative impact on the performance of FARM [80].

**Figure 10:**
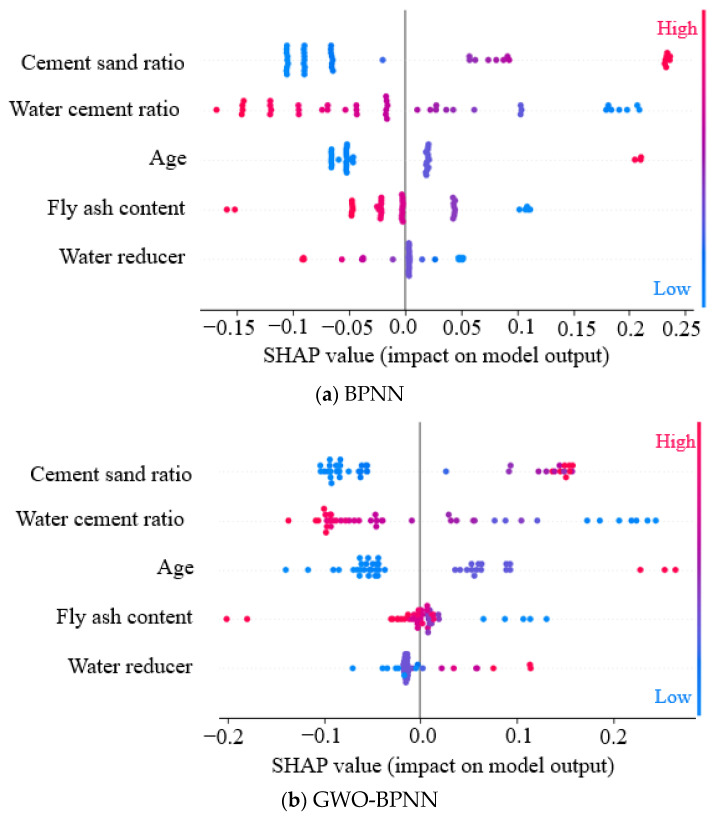
Global interpretations of the BPNN/GWO-BPNN model by SHAP values.

Figure 10 shows the distribution of SHAP values for each type of input parameter, which can demonstrate the importance of the input parameters and the direction of their impact on the output results. Different colors in the figure represent the magnitude of input parameter values, while the corresponding SHAP value on the horizontal axis indicates the impact of this input parameter on the output results, and the vertical axis represents the importance of input parameters [81]. It can be seen from Figure 10 that the cement/sand ratio has the greatest impact on the compressive strength of FARM, followed by the *w*/*c*, age, fly ash content, and water reducer. The distribution range of SHAP values of the FA content and water reducer is narrow and mainly concentrated around 0, indicating a weak relationship between the two and compressive strength. This result is consistent with the analysis of the feature heatmap in Figure 7. Comparing the SHAP graphs before and after optimization, the distribution range of SHAP values of the cement/sand ratio, *w*/*c,* and age obtained by the optimization model has widened, while that of the FA content and water reducer has narrowed. It indicates that the GWO-BPNN model has a more refined analysis of the relationship between input parameters and output results, indirectly verifying that the performance of the GWO-BPNN model is better than that of the BPNN model.

## 6. Conclusions

This article involved selecting six mix proportions of FARM with reasonable flowability to study the influence of *w*/*c* on compressive strength. The results showed that, under the same content of water reducer, a decrease in *w*/*c* resulted in an increase in compressive strength. Meanwhile, the GWO-BPNN model was established to predict the compressive strength of FARM and analyze the relation between its mix proportion and compressive strength. The data used for model training and validation are from the testing results of this paper and published studies. The model analysis results are given as follows:

(1) The performance of the GWO-BPNN model, compared to traditional BPNN models, significantly improved. After GWO optimization, *R*^2^ increased from 0.84 to 0.92, the MSE of the validation process decreased from 0.007 to 0.0015, and the iterations for convergence decreased from 108 to 65. This indicates that the GWO can effectively solve the problem of the BPNN becoming easily stuck in local minima, thereby improving the prediction accuracy and convergence speed of the model.
(2) The feature heatmap shows that the relationship between compressive strength and *w*/*c*, as well as the cement/sand ratio, is strongly negatively correlated and positively correlated, respectively, but the relationship with fly ash is highly uncertain. As seen from the graph of the joint plot, the published studies mainly focus on the mechanical properties of FARM with high *w*/*c*, which are greatly affected by *w*/*c*. The mechanical properties of FARM with low *w*/*c* are mainly from the experimental data in this article and are less affected by the *w*/*c*.
(3) The scatter matrix diagram further reveals that the compressive strength of FARM with a *w*/*c* higher than 0.5 is less than 20 MPa and that with a cement/sand ratio below 0.5 is typically below 25 MPa. The FA content in FARM is usually between 20% and 30%, while the content of the water reducer generally does not exceed 5% of the total cementitious material.
(4) The SHAP graph shows that the cement/sand ratio has the greatest impact on the compressive strength of FARM, followed by the *w*/*c* and age. The cement/sand ratio and age have a positive effect on compressive strength, while the *w*/*c* has a negative effect. The relationship between FA, the water reducer, and compressive strength is not strong. In addition, the distribution change in SHAP values before and after model optimization indicates that the GWO-BPNN model has a more refined analysis of the relationship between the input and output.

The GWO-BPNN was used to predict the compressive strength of FARM, providing an important reference for sustainable construction. However, many factors, such as the type, water absorption rate, the service environment of recycled aggregates, and its substitution rate in the mix proportion, which can affect the performance of FARM, did not need to be considered in this paper. In addition, the different models of machine learning for the strength prediction of FARM could be compared to identify optimal prediction algorithms. These works are challenging but beneficial for promoting the AI prediction of the performance of concrete materials.

## Figures and Tables

**Table 1:** Chemical composition of cement clinker (%).

Composition	SiO_2_	Al_2_O_3_	Fe_2_O_3_	CaO	MgO	SO_3_	Cl^−^
Content	24.99	8.26	4.03	51.42	3.71	2.51	0.043

**Table 2:** Properties of cement clinker.

Property	Fineness (0.08 mm Sieve Residue)/%	Water Requirement for Normal Consistency/%	Stability Boiling Method	Setting Time /min		Flexural Strength/MPa		Compressive Strength/MPa	
				**Initial**	**Final**	**3 d**	**28 d**	**3 d**	**28 d**
Result	2.9	28.2	Qualified	184	265	5.6	8.6	26.3	46.9

**Table 3:** Chemical composition of FA %.

Composition	MgO	Al_2_O_3_	SiO_2_	CaO	MgO	SO_3_	K_2_O	Fe_2_O_3_
Content	1.00	31.14	53.96	4.01	3.71	2.51	2.035	4.16

**Table 4:** Particle size screening of recycled fine aggregate.

Aperture Size	4.75 mm	2.36 mm	1.18 mm	0.6 mm	0.3 mm	0.15 mm
Accumulated amount of screening residue /%	14	33	57	78	100	100

**Table 5:** Mix proportions of FARMs with low *w*/*c* and their flowability.

No.	*w*/*c*	Cement/Sand Ratio	Cement/g	FA/g	Water/mL	Water Reducer/%	Fluidity/mm
1	0.26	0.89	700	300	260	2.5	210
2	0.25	0.89	700	300	250	2.5	195
3	0.24	0.89	700	300	240	3.0	170
4	0.23	0.89	700	300	230	3.0	165
5	0.22	0.89	700	300	220	4.0	165
6	0.2	0.89	700	300	200	4.0	150
7	0.22	0.89	700	300	200	3.5	145
8	0.2	0.89	700	300	200	3.5	145
9	0.2	0.89	700	300	200	3.0	130

**Table 6:** Data sets used in the GWO-BPNN model.

No.	Source	Number of Set	Proportion
1	Gao et al. [62]	3	2%
2	Li et al. [63]	9	6%
3	Mou et al. [64]	3	2%
4	Fu et al. [65]	4	3%
5	Liu et al. [66]	3	2%
6	Sui et al. [67]	81	54%
7	Kurad et al. [68]	20	14%
8	K et al. [69]	3	2%
9	The experiments conducted in this paper	24	15%

**Table 7:** Predictive results of compressive strength of FARM by the GWO-BPNN model.

No.	Actual Value (MPa)	Predictive Value (MPa)	Relative Error (%)	No.	Actual Value (MPa)	Predictive Value (MPa)	Relative Error (%)
1	4.2	4.27	1.6%	8	8.1	7.2	11.0%
2	5.6	5.39	3.5%	9	30.9	32.5	5.1%
3	2.7	2.47	8.5%	10	18.2	19.4	6.5%
4	30.9	31.5	1.9%	11	18.2	17.2	5.4%
5	37.4	36.6	2.1%	12	38.6	37.2	3.6%
6	34.5	33.8	2.0%	13	39.0	37.2	4.6%
7	8.9	10.1	2.2%	14	37.8	37.0	2.1%

## Data Availability

The original contributions presented in this study are included in the article. Further inquiries can be directed to the corresponding author.
